# Soluble Suppression of Tumorigenicity 2 (sST2) as a Diagnostic and Prognostic Marker in Acute Heart Failure and Sepsis: A Comparative Analysis

**DOI:** 10.3390/diagnostics15081010

**Published:** 2025-04-16

**Authors:** Flavio Davini, Marta Fogolari, Giorgio D’Avanzo, Maria Vittoria Ristori, Serena Nucciarelli, Lucrezia Bani, Antonio Cristiano, Marina De Cesaris, Silvia Spoto, Silvia Angeletti

**Affiliations:** 1 Research Unit of Clinical Laboratory Science, Department of Medicine and Surgery, Università Campus Bio-Medico di Roma, Via Alvaro del Portillo, 21, 00128 Roma, Italy; flavio.davini@unicampus.it (F.D.); serena.nucciarelli@unicampus.it (S.N.); 2 Operative Research Unit of Laboratory, Fondazione Policlinico Universitario Campus Bio-Medico, Via Alvaro del Portillo, 200, 00128 Rome, Italy; m.fogolari@policlinicocampus.it (M.F.); m.ristori@policlinicocampus.it (M.V.R.); lucreziabani3@gmail.com (L.B.); a.cristiano@policlinicocampus.it (A.C.); m.decesaris@policlinicocampus.it (M.D.C.); 3 Diagnostic and Therapeutic Medicine Departement, Fondazione Policlinico Universitario Campus Bio-Medico di Roma, Via Alvaro del Portillo, 200, 00128 Rome, Italy; giorgio.davanzo@policlinicocampus.it (G.D.); s.spoto@policlinicocampus.it (S.S.)

**Keywords:** biomarkers, ST2, suppression of tumorigenicity 2, diagnostic marker, prognostic marker, acute heart failure, sepsis

## Abstract

**Background:** Suppression of Tumorigenicity 2 (ST2), a member of the interleukin-1 receptor family, plays a crucial role in immune regulation. Elevated sST2 levels are associated with poor prognosis in various inflammatory and cardiovascular diseases, including acute heart failure (AHF), sepsis and transplant rejection. **Objectives and methods:** This study aimed to evaluate the diagnostic and prognostic accuracy of sST2, along with other biomarkers, such as high-sensitivity C-reactive protein (hs-CRP), N-terminal pro-B-type natriuretic peptide (NT-proBNP), procalcitonin (PCT) and mid-regional pro-adrenomedullin (MR-proADM), in patients with AHF, sepsis and AHF/sepsis overlap. **Results:** A cohort of 74 patients was analyzed, and comparison statistics revealed that sST2 levels were significantly higher in the AHF/sepsis group (113.88 ng/mL) compared to the AHF group (42.24 ng/mL, *p* = 0.024), while no significant difference was observed between sepsis and AHF groups (*p* = 0.10). Other biomarkers, including hs-CRP and PCT, showed significant differences between the AHF and AHF/sepsis groups. ROC curve analysis identified sST2 as a strong predictor of mortality and readmission, with high AUC values for 30-day readmission (0.821) and mortality (0.87). **Conclusions:** These findings suggest that combining biomarkers, including sST2, could improve the early diagnosis, risk stratification and management of critically ill patients with overlapping AHF and sepsis. Further studies with larger populations are needed to validate these findings and explore the potential of integrating these biomarkers into clinical practice.

## 1. Introduction

Suppression of Tumorigenicity 2 (ST2) is a protein belonging to the interleukin-1 receptor (IL-1R) family and plays a pivotal role in immune regulation. It is expressed in two primary forms due to alternative splicing: a transmembrane receptor form, known as ST2L, and a soluble truncated form, referred to as sST2. sST2 is secreted into the bloodstream, where it acts as a “decoy” receptor [1]. sST2 has emerged as a significant prognostic biomarker in the field of cardiovascular disease, particularly noted for its utility in risk stratification and the management of acute heart failure (AHF) [2]. Elevated levels of sST2 are strongly associated with worse clinical outcomes in AHF, including increased mortality and hospitalization rates. This makes sST2 a valuable tool in tailoring treatment strategies and monitoring disease progression [2,3,4]. Beyond its role in cardiovascular conditions, sST2 is also markedly increased in a variety of acute and chronic inflammatory diseases and pulmonary conditions, such as acute respiratory distress syndrome (ARDS) and chronic obstructive pulmonary disease (COPD), where it correlates with disease severity. Several studies have also demonstrated its role as a diagnostic and prognostic marker in patients with sepsis and septic shock. The measurement of sST2 and procalcitonin (PCT) in combination has been identified as useful for risk stratification and prognosis prediction in patients with suspected sepsis. These findings underscore the growing recognition of sST2 not only as a cardiovascular biomarker but also as a versatile indicator of systemic and localized inflammatory processes, offering valuable insights for diagnosis, prognosis and therapeutic monitoring across various medical disciplines [5,6,7,8,9,10,11]. Although the performance of sST2 as a marker in cardiovascular pathology has already been previously examined by the scientific literature, its role in severely hospitalized patients with often complex clinical conditions is still under investigation. AHF can often complicate other serious conditions, as seen in sepsis associated with organ damage (SAOD). Conversely, the onset of an infectious condition on top of a pre-existing acute cardiac disease can significantly worsen and compromise the prognosis of already critically ill patients. The combined use of several diagnostic and prognostic markers may help in the early detection of patients with sepsis and acute heart failure and support the stratification of disease severity in order to rapidly implement appropriate therapeutic interventions to improve the outcome of critically ill in-patients [12,13,14]. This prospective observational pilot study was designed to assess the combined diagnostic and prognostic accuracy of sST2 plasma markers, such as highly sensitive C-Reactive Protein (CRP), N-terminal pro–B-type natriuretic peptide (NT-ProBNP), PCT and mid-regional pro-adrenomedullin (MR-proADM), in patients diagnosed with AHF or sepsis/septic shock. In this context, the use of a multi-marker approach is essential to improve diagnostic efficiency and provide prognostic evaluations. The integration of biomarkers provides complementary information from a single blood sample to the clinician, offering a significant advantage in clinical management. It provides an opportunity to offer the patient the best possible treatment in the shortest time.

## 2. Materials and Methods

Seventy-four patients hospitalized at the Diagnostic and Therapeutic Medicine Department of the Fondazione Policlinico Universitario Campus Bio-Medico of Rome from 1 July 18 to 31 July 2023 were included in the present study. Of these, 57/74 (77%) had AHF, 8/74 (11%) had sepsis and 9/74 (12%) were diagnosed with overlapping AHF and sepsis. The degree of heart failure in patients included in the study was classified using the NYHA. The diagnosis of sepsis was performed according to the guidelines of the Third International Consensus Definitions for Sepsis and Septic Shock (Sepsis-3) and the diagnosis of congestive heart failure was based on clinical assessment accompanied by correlation with biomarkers and imaging. All patients underwent blood sampling for the markers under study at the time of ward admission. The follow-up of the enrolled patients was clinical and was continued until their discharge.

Inclusion and exclusion criteria were as follows. Patients were enrolled if they were aged ≥ 18 years and if they had received diagnosis of AHF and/or sepsis by an internal medicine physician or the emergency department. Patients were excluded from the study in cases of pregnancy, evidence of acute coronary syndrome and if unable to comply with protocol due to psychiatric disease.

### 2.1. ST2 Testing

At enrollment, patient blood samples were collected in plastic tubes with EDTA anticoagulant, and plasma was isolated within 60 min of sample acquisition. Plasma samples were stored in plastic cryovials at −20 °C or lower. The soluble ST2 was measured using a fluorescence-based lateral flow immunoassay method via the AFIAS-1 platform (Boditech Med Inc., Gangwon, Republic of Korea). sST2 was measured also in cases of readmission at 30, 60 and 90 days.

### 2.2. Biomarker Measurement

Plasma concentrations of NT-proBNP, CRP and PCT were measured via the chemiluminescence method using Alinity I (Autoanalyzer Abbott). The blood concentrations of MR-proADM were measured with an automatic Kryptor analyzer, using a time-resolved amplified emission method (Kryptor; Brahms AG; Hennigsdorf, Germany) with commercially available immunoassays. Plasma biomarkers were measured at admission, and in cases of readmission at 30, 60 and 90 days.

### 2.3. Statistical Analysis

The data obtained were expressed as the median and interquartile range and were statistically evaluated with median and Mann–Whitney test, to compare sST2, CRP, NT-proBNP, PCT and MR-proADM concentrations between the three groups of AHF, AFH/sepsis and sepsis, providing a robust, non-parametric way to compare independent samples, two at a time. *p*-value ≤ 0.05 was considered statistically significant.

## 3. Results

A table summarizing patients’ baseline characteristics is included (Table 1). The study population consisted of 67 survivors and 7 non-survivors.

The studied patient population appears to be fairly homogeneous in terms of age and gender, with no statistically significant differences between survivors and non-survivors. Regarding the comorbidities considered, the prevalence of hypertension in the survivors group was statistically significant (*p* = 0.011), as was the presence of reduced ejection fraction (EF < 40%) in the non-survivors group (*p* = 0.020). In terms of AHF patients, based on the NYHA classification they were defined as follows: NYHA 1 in 12/66 (17%), NYHA 2 in 25/66 (38%), NYHA 3 in 15/66 (23%) and NYHA in 14/66 (22%).

The underlying conditions considered risk factors for AHF and sepsis development were, respectively, diabetes, hypertension, atrial fibrillation, hypertensive heart disease, ischemic heart disease, cerebrovascular disease and atherosclerotic disease for AHF and diabetes, COPD (chronic obstructive pulmonary disease), neoplasia and chronic renal failure for sepsis. 

Median values of the selected biomarkers are reported in Table 2.

**Figure 1:**
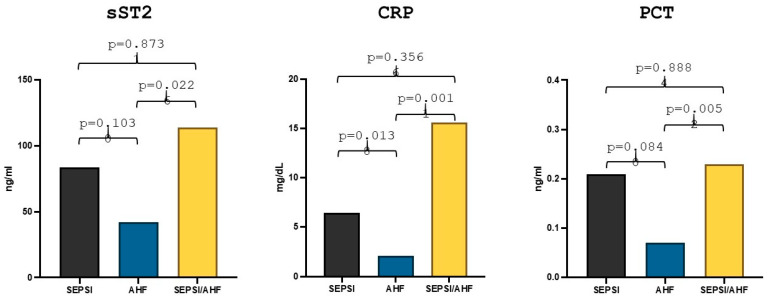
Differences in sST2, hs-CRP and PCT values in acute heart failure, sepsis and AHF/sepsis groups. Significant *p*-value ≤ 0.05.

Comparison statistics showed that in the group of patients with acute heart failure (57 patients) the median sST2 value was 42.24 ng/mL, whereas in the group of patients with sepsis and acute heart failure (9 patients) the median value was 113.88 ng/mL, the difference between the two groups reached statistical significance (*p* = 0.024). Moreover, in the group of patients with sepsis (8 patients), the median sST2 value was 83.84 ng/mL, but there were no statistically significant differences in the values compared to the group of patients with AHF (*p* = 0.10) (Figure 1). With regard to the other markers, the median high-sensitivity CRP value in the group of patients with AHF was 2.08 mg/dL while the median value in the AHF/sepsis group was 15.57 mg/dL and the median value of high-sensitivity CRP in the group of patients with sepsis was 6.45 mg/dL. The difference was statistically significant both between the AHF group and the AHF/sepsis group (*p* = 0.019) and between the AHF group and the sepsis group (*p* = 0.016) (Figure 1).

PCT median value in the AHF group was 0.07 ng/mL while in the AHF/sepsis group PCT value was 0.23 ng/mL and the statistical comparison between the two groups showed a statistically significant difference (*p* = 0.0056). The median value of PCT in the sepsis group was 0.21 ng/mL but the difference with the group of patients with AHF remained statistically non-significant (*p* = 0.084) (Figure 1).

**Figure 2:**
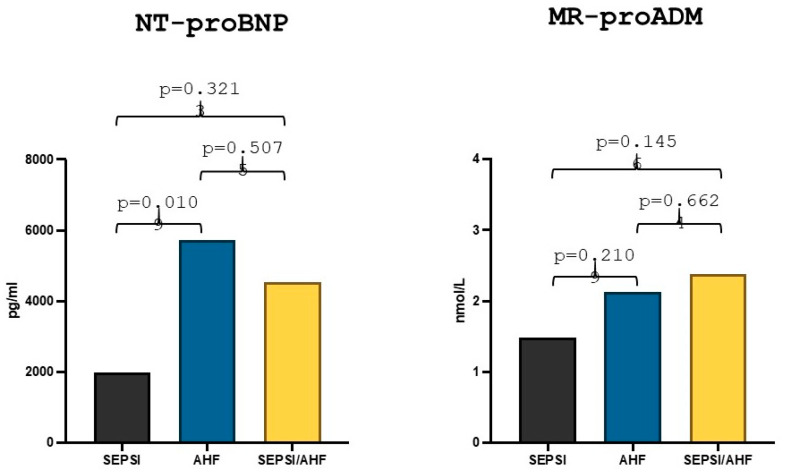
Differences in NT-proBNP and MR-proADM values in acute heart failure, sepsis and AHF/sepsis groups. Significant *p*-value ≤ 0.05.

The median value of NT-proBNP in the AHF group was 5727 pg/mL, while the median value in the group with AHF/sepsis was 4544 pg/mL and the comparative statistical analysis between the two groups showed no statistically significant differences (*p* = 0.507). The median value of NT-proBNP in the sepsis group was 1974.5 pg/mL, and there was a statistically significant difference between the values in the AHF group in comparison to the sepsis group (*p* = 0.010) (Figure 2). Lastly, the MR-proADM median value in the AHF group was 2.13 nmol/L while the median value in the AHF/sepsis group was 2.38 nmol/L and the statistical comparison showed no statically significant differences (*p* = 0.66). The median value of MR-proADM in the sepsis group was 1.48 nmol/L and there was no statistically significant difference with the AHF group (*p* = 0.21) (Figure 2).

**Figure 3:**
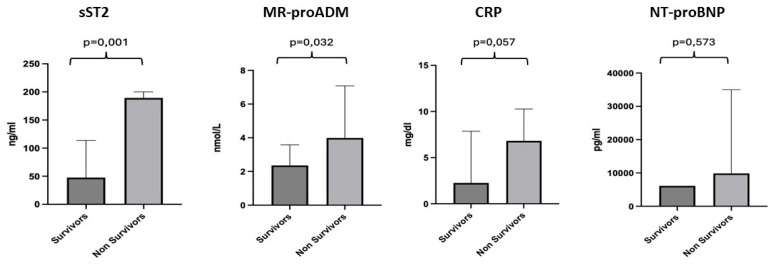
Differences in the values of sST2, MR-proADM, CRP and NT-proBNP between the survivor and non-survivor groups. Significant *p*-value ≤ 0.05.

We conducted a more detailed analysis of the individual biomarkers by dividing the population into two groups: survivors (67 patients), with a median age of 82 years, and non-survivors (7 patients), with a median age of 81 years. For sST2, a median value of 47.66 ng/mL was observed for the survivor subgroup and 189.4 ng/mL for the non-survivors, with a *p*-value of 0.001. For MR-proADM, a median value of 2.25 nmol/L was observed for the survivor subgroup and 3.99 nmol/L for the non-survivors, with a *p*-value of 0.032. CRP showed a median value of 2.26 mg/dl for the survivor subgroup compared to 6.81 mg/dl for the non-survivors, with a *p*-value of 0.057. For NT-proBNP, we found a median value of 6143 pg/mL for the survivors and 9882 pg/mL for the non-survivors, with no statistically significant difference, *p*-value = 0.573 (Figure 3).

**Figure 4:**
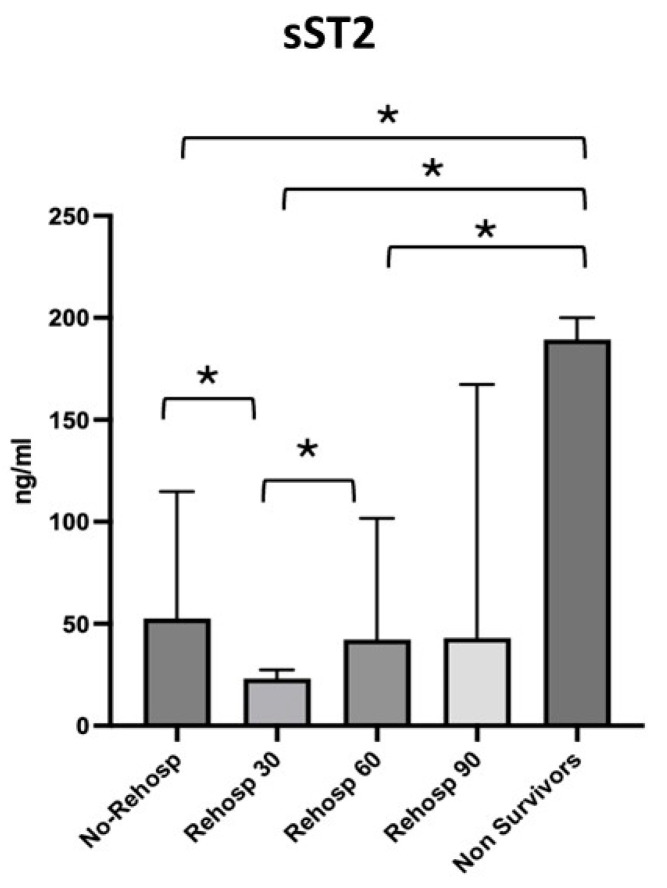
Differences in sST2 values across the subgroups of survivors and non-survivors. Significant *p*-value ≤ 0.05. Square brackets with an asterisk indicate statistically significant differences between groups.

The four subgroups identified were compared with each other and with the non-survivor group, for which the highest values of sST2 were observed. The first group, consisting of patients with no need for readmission, showed a median sST2 value of 52.62 ng/mL [27.95–114.8]; the second group, consisting of patients with readmission at 30 days, showed a median value of 23.17 ng/mL [13.69–27.43]; the third group, consisting of patients with readmission at 60 days, showed a median value of 42.24 ng/mL [40.18–101.7]; and finally, the last group of patients with readmission at 90 days showed a median value of 43.01 ng/mL [9.53–167.3] (Figure 4).

Lower sST2 median values were observed in the group of patients who were not readmitted compared to the group of patients readmitted at 30 days (*p* = 0.021), whereas higher levels were seen in the group of patients readmitted at 30 days compared to those readmitted at 60 days (*p* = 0.007). No significant variations in sST2 were observed between readmissions at 60 and 90 days. The value of sST2 in survivors was significantly higher compared to the group of non-readmitted patients (*p* = 0.001), the group readmitted at 30 days (*p* = 0.002) and the group readmitted at 60 days (*p* = 0.010) but not compared to the group readmitted at 90 days (*p* = 0.101).

**Figure 5:**
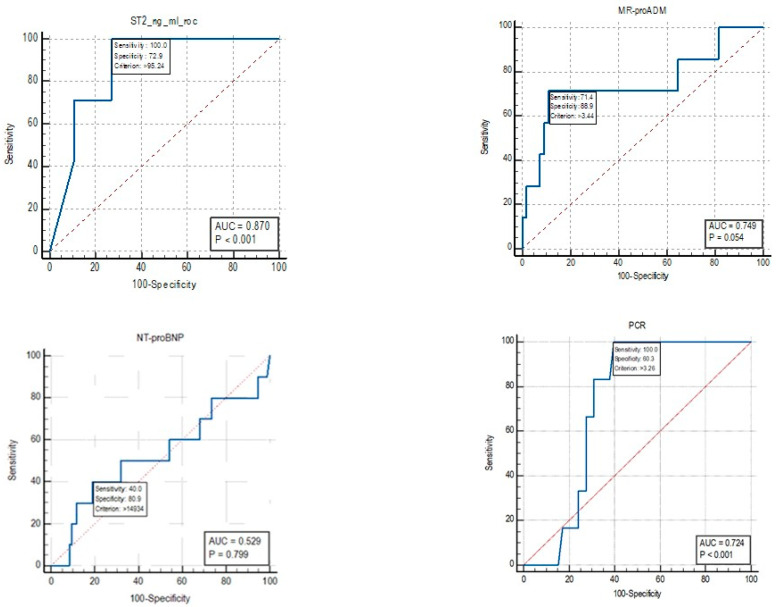
ROC curve for the biomarkers sST2, MR-proADM, CRP and NT-proBNP.

For mortality, the cut-off identified by the ROC curve for sST2 was 95.24 ng/mL (Figure 5). An Area Under Curve (AUC) value of 0.87 was identified, with a sensitivity of 100% and a specificity of 72.9%. For the biomarker MR-proADM, an AUC value of 0.749 was identified, with a sensitivity of 71.4% and a specificity of 88.9% for mortality cut-off values of >3.44 nmol/L.

The AUC for NT-proBNP was 0.529, and therefore, a cut-off for mortality was not reported as the test is not informative.

For the CRP biomarker, an AUC value of 0.724 was identified, with a sensitivity of 100% and a specificity of 60.3% for mortality cut-off values of >3.26 nmol/L.

**Figure 6:**
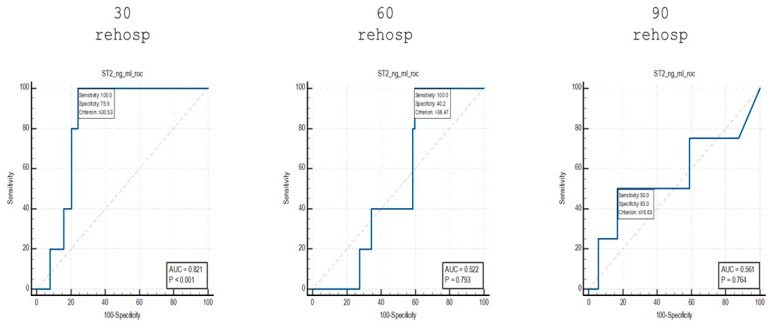
ROC curve analysis of sST2 values in patients who were readmitted at different time points.

To highlight the differences identified in the stratification of patients based on the risk of readmission, an analysis was performed using ROC curves of the sST2 values identified among the subgroups of patients who were readmitted at 30, 60 and 90 days (Figure 6).

When comparing patients who were readmitted at 30 days to non-readmitted patients, an AUC value of 0.821 was identified, with a sensitivity of 100% and a specificity of 75.9% for cut-off values of ≤30.53 ng/mL.

Using the same analytical approach, for patients who were readmitted at 60 days, an AUC value of 0.522 was identified, with a sensitivity of 100% and a specificity of 40.2% for cut-off values of >38.47 ng/mL.

Finally, for the group of patients who were readmitted at 90 days, an AUC value of 0.561 was identified, with a sensitivity of 50% and a specificity of 83% for cut-off values of ≤16.63 ng/mL.

## 4. Discussion

In our study, sST2 levels mirrored the trends observed with high-sensitivity C-reactive protein (hs-CRP), with both markers significantly elevated in the acute heart failure (AHF)/sepsis group compared to the AHF or sepsis groups individually. This observation underscores the potential of sST2 and hs-CRP as biomarkers that reflect the interplay between inflammatory and cardiovascular processes. Both markers are significantly elevated in the AHF/sepsis group, suggesting a potential shared background of inflammation. Similar findings have been reported in other studies, supporting the robustness of our observations. For example, Jenkins et al. [15] demonstrated elevated sST2 levels in patients with sepsis and septic shock, correlating them with disease severity and adverse outcomes, while hs-CRP was similarly linked to heightened inflammation across cardiac and non-cardiac pathologies. Higher sST2 levels correlate with worse outcomes, including increased mortality rates [16]. Another study reported that patients with persistent or increasing sST2 levels during recovery had a 37.5% one-year mortality rate [17]. hs-CRP is an established inflammatory marker, and its elevation alongside sST2 indicates a heightened inflammatory response in AHF and sepsis [18]. Both biomarkers can serve as independent risk factors for adverse outcomes, enhancing risk stratification in clinical settings [16]. When evaluating PCT, its role as a sepsis-specific biomarker has been widely validated in the literature. A meta-analysis by Wacker et al. [19] highlighted the diagnostic accuracy of PCT in distinguishing sepsis from other non-infectious inflammatory conditions, reporting a pooled sensitivity of 77% and a specificity of 79%. Our findings align with this paradigm, showing a marked increase in PCT levels in both the sepsis and AHF/sepsis groups. However, the statistically significant difference observed only between the AHF and AHF/sepsis groups, but not between the sepsis and AHF groups, may suggest a more nuanced interplay between systemic infection and cardiac dysfunction. While sST2 and hs-CRP are promising biomarkers, their clinical utility may vary based on individual patient factors and the complexity of underlying conditions, necessitating further research to optimize their application in diverse clinical scenarios. The findings regarding PCT levels in sepsis and AHF suggest a complex relationship between systemic infection and cardiac dysfunction. The significant difference in PCT levels between AHF and AHF/sepsis groups, but not between sepsis and AHF groups, indicates that AHF may influence PCT responses differently. This complexity may be exacerbated by the limited sample size, which can obscure significant trends, as noted in other studies with small populations [20,21]. PCT is recognized for its diagnostic and prognostic value in sepsis, with studies showing elevated levels in septic patients compared to healthy controls [22]. The utility of PCT in distinguishing between sepsis and AHF remains debated, with some studies indicating overlapping PCT levels in these conditions [20]. Bouhemad et al. [23] investigated the intricate link between sepsis and cardiac biomarkers, revealing that sepsis-induced myocardial dysfunction contributes to the elevation of markers such as PCT and sST2. Similarly, studies on the prognostic value of sST2 in acute heart failure have shown its association with systemic inflammation and adverse cardiac remodeling, as highlighted by Dieplinger et al. [24]. These findings reinforce the need for larger cohort studies to validate the interplay of these markers in overlapping syndromes like AHF/sepsis [25]. Notably, the absence of a significant difference in PCT values between the sepsis and AHF groups in our study may be attributed to the limited sample size. Indeed, limited sample sizes can mask significant differences in biomarker levels, as variability within clinical phenotypes may lead to inconclusive results [21]. Similar challenges have been reported in studies with small populations, where the variability within clinical phenotypes can mask statistically significant trends. Contrary to other markers, NT-proBNP levels in our study did not exhibit statistically significant differences between the AHF and AHF/sepsis groups. This aligns with its established role in diagnosing and managing congestive heart failure rather than distinguishing between overlapping conditions like AHF and sepsis. Studies such as those by Maisel et al. [26] have consistently shown NT-proBNP to be a reliable biomarker for heart failure diagnosis, correlating strongly with ventricular wall stress and volume overload. Interestingly, median NT-proBNP levels in our sepsis group were significantly lower than in the AHF and AHF/sepsis groups. This finding corroborates prior studies, such as Zhang et al. [27], which observed relatively low NT-proBNP levels in sepsis patients without direct cardiac involvement. Nevertheless, elevated NT-proBNP in sepsis has been associated with sepsis-induced myocardial dysfunction (SIMD), as described by Bouhemad et al. [23]. The absence of significant differences in NT-proBNP between the AHF and AHF/sepsis groups in our study may reflect overlapping cardiac stress mechanisms rather than distinct inflammatory or infectious contributions. This study’s findings regarding NT-proBNP levels highlight its established role in diagnosing heart failure (HF) rather than differentiating between AHF and sepsis. The lower median NT-proBNP levels observed in the sepsis group compared to AHF and AHF/sepsis groups suggest that NT-proBNP may not be a reliable marker in overlapping conditions. This aligns with the existing literature that emphasizes the importance of context when interpreting NT-proBNP levels [28,29,30].

Similarly, MR-proADM levels in our study did not show statistically significant differences across the three groups examined. This might reflect its broader role as a prognostic rather than diagnostic marker, particularly in predicting disease severity and mortality risk. Studies by Elke et al. [31] and Latini et al. [32] have emphasized MR-proADM’s superior prognostic value compared to traditional biomarkers like NT-proBNP, hs-CRP and PCT, particularly in predicting 30-day mortality in septic and heart failure patients. Our findings about MR-proADM levels in AHF and AHF/sepsis groups compared to the sepsis group align with its established role in cardiovascular stress and decompensation. Moreover, MR-proADM’s capacity to predict mortality and organ failure in mixed populations of critically ill patients, as highlighted by Saeed et al. [33], underscores its utility in stratifying patients at high risk, particularly in those with overlapping AHF/sepsis syndromes.

The absence of significant differences in MR-proADM across groups in our study may also be attributable to the clinical fragility and severity of illness among our patients, factors that are known to universally elevate MR-proADM levels [34].

Our preliminary research highlights the potential utility of sST2 as a marker of inflammation and clinical severity in patients with sepsis and in those with overlapping AHF and sepsis. This finding aligns with prior studies that have demonstrated sST2’s role as a dynamic biomarker reflecting systemic inflammation, cardiac stress and poor outcomes in critically ill patients. For example, the study by Januzzi et al. [35] emphasized the prognostic value of sST2 in acute heart failure, where elevated levels were associated with worse outcomes, including increased mortality and hospital readmissions. Similarly, Gruden et al. [36] reported sST2 elevations in sepsis, correlating with disease severity and organ dysfunction.

The combined use of biomarkers such as sST2, hs-CRP and PCT holds promise for improving the early identification and management of patients with overlapping syndromes like AHF and sepsis. Each biomarker offers unique insights. For instance, sST2 reflects systemic inflammation and myocardial stress, offering value in both cardiac and inflammatory conditions. Its dynamic responsiveness to therapeutic interventions further supports its utility in monitoring clinical progression. hs-CRP is a well-established marker of systemic inflammation, with elevated levels indicating heightened inflammatory states. According to Ridker et al., hs-CRP effectively stratifies cardiovascular risk and reflects inflammatory burden in infectious and non-infectious contexts [37]. PCT has been extensively validated for its role in sepsis, as demonstrated by Wacker et al. in a meta-analysis showing its diagnostic accuracy in distinguishing bacterial infections from other inflammatory states [19].

In this study, a detailed analysis of the biomarkers was performed dividing the population into two groups: survivors (67 patients) and non-survivors (7 patients).

The results indicate significant differences in the biomarkers between survivors and non-survivors. For sST2 and MR-proADM, non-survivors exhibited a markedly higher median value, suggesting that both biomarkers could be good predictor of mortality [38,39]. Although CRP showed a trend toward higher values in non-survivors (6.81 mg/dL vs. 2.26 mg/dL in survivors), the *p*-value of 0.057 did not reach statistical significance [40]. NT-proBNP did not show any significant difference between the two groups (*p*-value 0.573), indicating that it may not be a reliable marker for predicting mortality in this sample [41].

In a further analysis of the subgroups of survivors based on readmission, sST2 levels were again found to be informative. The group of patients who were not readmitted had a lower median sST2 value (52.62 ng/mL) compared to those readmitted at 30 days (23.17 ng/mL), with the latter group having significantly higher sST2 levels (*p* = 0.021). Higher levels of sST2 were also observed in patients readmitted at 30 days compared to those readmitted at 60 days (*p* = 0.007). However, no significant variation in sST2 levels was seen between patients readmitted at 60 and 90 days. Interestingly, survivors exhibited higher sST2 levels than those in the non-readmitted group, as well as the 30-day and 60-day readmission groups, but not compared to the 90-day readmission group [18].

The ROC curve analysis for mortality prediction revealed promising results for sST2, which showed an Area Under Curve (AUC) value of 0.87 with a sensitivity of 100% and a specificity of 72.9% for a cut-off value of 95.24 ng/mL. MR-proADM also showed useful performance with an AUC of 0.749, a sensitivity of 71.4% and a specificity of 88.9% for a mortality cut-off value of >3.44 nmol/L. In contrast, NT-proBNP did not show any significant predictive value for mortality (AUC = 0.529), while CRP demonstrated moderate diagnostic performance with an AUC of 0.724, a sensitivity of 100% and a specificity of 60.3% for cut-off values of >3.26 nmol/L.

Furthermore, the ROC curve analysis for readmission prediction demonstrated that sST2 was able to differentiate between patients who were readmitted at 30 days and those who were not readmitted, with an AUC of 0.821, sensitivity of 100% and specificity of 75.9% for cut-off values of ≤30.53 ng/mL. However, sST2 was less effective in predicting readmissions at 60 and 90 days, with AUC values of 0.522 and 0.561, respectively, indicating weaker predictive capacity as the time to readmission increased.

Notably, the multi-marker approach could be particularly valuable in the early identification and stratification of patients with AHF/sepsis, a population at heightened risk of rapid clinical deterioration, as demonstrated by other authors [42,43,44].

A limitation of the study lies in the small number of patients examined. This study, in fact, represents a pilot study intended to initiate analyses on larger groups of patients. However, we deemed it useful to share these preliminary results because we believe the multi-marker approach is highly valuable. In this approach, various biomarkers are compared, and by integrating them, they provide a holistic view that offers significant support for clinical decision-making, not only for diagnostic purposes but also for prognostic ones. Identifying, even in a limited cohort of patients, a certain degree of significance is promising and could serve as a starting point for larger, multicenter studies.

## 5. Conclusions

Data from this preliminary study suggest that the multi-marker approach could be particularly valuable in the early identification and stratification of patients with AHF/sepsis, a population at heightened risk of rapid clinical deterioration, as demonstrated by other authors [42,43,44]. Overall, the results highlight the importance of sST2 as a potential biomarker for predicting both mortality and the risk of readmission within 30 days. MR-proADM also emerged as a valuable marker, whereas NT-proBNP showed limited utility in this cohort. The findings suggest that monitoring sST2 levels could help in identifying high-risk patients, potentially guiding clinical decision-making in managing individuals at risk for adverse outcomes such as mortality and readmission.

We deemed it useful to share these preliminary results based on this approach, where various biomarkers were compared and integrated. This approach provides a holistic view offering significant support for clinical decision-making, not only for diagnostic purposes but also for prognostic ones. A limitation of the study lies in the small number of patients examined. This research, in fact, represents a pilot study intended to initiate analyses in larger groups of patients. Identifying, even in a limited cohort of patients, a certain degree of significance is promising and could serve as a starting point for larger, multicenter studies.

Further studies should explore how these biomarkers interact in diverse populations, examining their combined diagnostic and prognostic performance. The incorporation of novel technologies, such as machine learning algorithms for biomarker pattern recognition, may further enhance early identification and personalized management strategies for these high-risk patients.

## Figures and Tables

**Table 1:** Demographic characteristics and comorbidities of the study population divided into the two groups: survivors (67 patients) and non-survivors (7 patients).

	Total (74 Patients)	Survivors (67 Patients)	Non-Survivors (7 Patients)	*p*-Value *
Male (%)	43 (58)	34 (50.6)	6 (85.7)	0.078
Female (%)	31 (42)	33 (49.4)	1 (14.3)	0.078
Median age [IQR 25–75]	82 [73–88]	82 [72–88]	81 [78–84]	0.741 **
Hypertension (%)	65 (88)	61 (90.6)	4 (57.1)	0.011
Diabetes (%)	22 (29.3)	20 (29.4)	2 (28.6)	0.965
Hyperlipidemia (%)	26 (34.8)	25 (36.5)	1 (14.3)	0.242
Smoking (%)	26 (34.8)	24 (35.3)	2 (28.6)	0.724
Atrial Fibrillation (%)	41 (55.4)	29 (57.6)	2 (28.6)	0.145
Hypertensive Cardiopathy (%)	22 (29.3)	20 (29.4)	2 (28.6)	0.965
Hypertensive-Degenerative Cardiopathy (%)	39 (52.2)	35 (52.9)	3 (42.9)	0.616
Ischemic Heart Disease (%)	23 (31.5)	22(32.9)	1 (14.3)	0.315
Cerebrovascular Disease (%)	26 (34.8)	25 (36.5)	1 (14.3)	0.242
Atherosclerotic Disease (%)	20 (27.2)	18 (27.1)	2 (28.6)	0.932
Chronic Obstructive Pulmonary Disease (COPD) (%)	26 (34.8)	24 (35.3)	2 (28.6)	0.724
Neoplasia (%)	14 (18.5)	11 (16.5)	3 (42.9)	0.092
Chronic Renal Failure (%)	49 (66.3)	45 (67.1)	4 (57.1)	0.597
Preserved EF (≥50%)	35 (46.7)	33 (49.4%)	1 (24.3%)	0.192
Mildly Reduced EF (between 49 and 41)	7 (9.8)	7 (10.6%)	0	-
Reduced EF (≤40%)	32 (43.5)	27 (40%)	6 (85.7%)	0.020
Total Readmissions	11 (15.2)	11 (16.5)	na	-
Readmission at 30 days (%)	4 (5.4)	4 (5.9)	na	-
Readmission at 60 days (%)	4 (5.4)	4 (5.9)	na	-
Readmission at 90 days (%)	3 (4.3)	3 (4.7)	na	-
Not Readmitted (%)	57 (77.2)	56 (83.5)	na	-
Median LOS [IQR 25–75]	10 [7–16.5]	10 [7–15]	15 [9.5–22]	0.368 **

* *p*-value refers to the comparison of survivors vs. non-survivors; IQR = interquartile range; LOS = length of stay. ** Mann–Whitney comparison.

**Table 2:** Biomarkers median values measured in blood of patients enrolled for the study.

Variables	Patients with AHF N = 57	Patients with Sepsis and AHF N = 9	Patients with Sepsis N = 8
sST2 (ng/mL) [IQR 25–75]	42.24 [16.68–102.77]	113.88 [57.54–189.38]	83.84 [62.09–197.66]
hs-CRP (mg/dL) [IQR 25–75]	2.08 [0.64–4.84]	15.57 [4.52–39.98]	6.45 [5.59–9.69]
NT-proBNP (pg/mL) [IQR 25–75]	5727 [2304.50–11343]	4544 [1653.27–13817.75]	1974.5 [1067.50–2441.50]
PCT (ng/mL) [IQR 25–75]	0.07 [0.03–0.2]	0.23 [0.13–1.37]	0.21 [0.21–0.67]
MR-proAM (nmol/L) [IQR 25–75]	2.13 [1.30–2.68]	2.38 [1.76–2.62]	1.48 [1.36–2.50]

## Data Availability

Data are unavailable due to privacy and ethical restrictions.

## Abbreviations

The following abbreviations are used in this manuscript:

ST2	Suppression of Tumorigenicity 2
IL-1R	Interleukin-1 receptor
IL-33	Interleukin-33
sST2	Soluble Suppression of Tumorigenicity 2
AHF	Acute heart failure
ARDS	Acute respiratory distress syndrome
COPD	Chronic obstructive pulmonary disease
IBD	Inflammatory bowel disease
PCT	Procalcitonin
SAOD	Sepsis associated with organ damage
hs-CRP	High-sensitivity C-reactive protein
NT-ProBNP	N-terminal pro-B-type natriuretic peptide
MR-proADM	Mid-regional pro-adrenomedullin
HF	Heart failure
